# Determinants of COVID-19 Vaccine Uptake and Acceptability in the Horn of Africa: Evidence from Somaliland

**DOI:** 10.3390/vaccines10071076

**Published:** 2022-07-04

**Authors:** Saed A. Sulub, Mubarik A. M. Igal

**Affiliations:** 1 School of Graduate Studies, University of Hargeisa, Hargeisa 25263, Somaliland; 2 School of Medicine and Health Sciences, Gollis University, Hargeisa 25263, Somaliland; mubarak7132@gmail.com; 3 Alif Research and Data Analytics Academy (ARDAA), Hargeisa 25263, Somaliland

**Keywords:** COVID-19 disease, SARS-CoV-2 vaccine, vaccine hesitancy, vaccine acceptance, willingness

## Abstract

Various COVID-19 vaccines have been developed in an unprecedented time and were rolled out across the world to save lives during the COVID-19 pandemic. Yet statistics show that COVID-19 uptake and acceptance in the Horn of Africa have been very low. To examine factors associated with COVID-19 vaccine uptake and acceptance in Somaliland, we carried out a cross-sectional study using a structured questionnaire both in offline and online modes. The study population was adults from the age of 18 years and above. Of the 704 respondents who participated in the survey, only 37% took the vaccine. Surprisingly, about 65% of those who have not taken the vaccine were unwilling to get vaccinated. Using a binomial logistic regression, we find that older people, the more educated and those who are employed are more likely to take the vaccine. Our results also show that the perceived threat of the virus and the perceived safety of the vaccine significantly increase vaccine uptake and acceptance. Results of this study will help the government and other concerned parties shape policies that can boost vaccine uptake and acceptance.

## 1. Introduction

As of 26 April 2022, there were over 500 million COVID-19 confirmed cases worldwide with over 6 million deaths recorded [1]. Aside from the loss of human lives, the pandemic also continues to disastrously affect the global economy [2]. To lessen its impact, a lightning fast quest for COVID-19 vaccines were initiated to combat this public health threat. Only a year into this tragedy, vaccines were introduced and authorized for use by international health regulatory bodies.

According to WHO, cases are in excess of 26,000, with over 1000 deaths in Somalia [1]. However, with the stigma surrounding COVID-19 and the lack of access to healthcare in many areas, there are many unreported cases and deaths. In fact, Uyoga et al. [3] studied seroprevalence among blood donors in Kenya and indicated that exposure to COVID-19 in Kenya is much higher than the reported cases. In an effort to reduce the burden of COVID-19 worldwide and ensure equitable access to COVID-19 vaccines, the COVID-19 Vaccines Global Access (COVAX) initiative delivered 300,000 doses of the AstraZeneca vaccine to Somalia in mid-March, 2021. Out of the number of doses delivered in the first batch, only 65,000 doses arrived in Hargeisa, the capital city of Somaliland [4]. It was then rolled out across all regions of Somaliland, and the vulnerable groups including the healthcare workers; the elderly; and those with underlying diseases such as diabetes, heart disease, hypertension, and obesity were prioritized. Other batches arrived at later dates, and the vaccine is currently accessible to all the population.

According to the latest statistics on the coronavirus vaccine, more than half of the world’s population (59.5%) is fully vaccinated [5]. Despite this high vaccination rate globally, a huge gap exists in the vaccination rates of different countries. Horn of Africa countries have the lowest vaccination rates, with less than 20% fully vaccinated [5]. Though an overly optimistic study in Somalia by Ahmed et al. [6] estimated that an overwhelming majority of the population (76.8%) intended to take the vaccine, only a minor fraction (8.7%) took it [5]. Moreover, while, at the moment, there are no publicly available statistics on vaccination rates in Somaliland, our survey of more than 700 respondents currently living in Somaliland has also shown that only a small number of people received the vaccine (36.56%).

Previous studies in the literature linked vaccine uptake and acceptability to sociodemographic factors and psychological factors. Among the sociodemographic factors that were consistently found to be significant in explaining vaccine uptake and willingness to get vaccinated are age (e.g., Machida et al., [7]; Malik et al., [8]; Dula et al., [9]; Soares et al., [10]; Robinson et al., [11]) and education (e.g., Machida et al., [7]; Malik et al., [8]; Robinson et al., [11] Chaudhary et al., [12]; Lazarus et al., [13]). A significant number of studies have also provided evidence of other factors that are associated with a higher likelihood of COVID-19 vaccine acceptance and uptake such as income (e.g., Machida et al., [7]), being a healthcare worker (e.g., Ahmed et al., [6]), and the presence of underlying chronic health conditions (e.g., Machida et al., [7]; Dula et al., [9]; Gruner & Kruger., [14]). In this regard, it is important to note that the literature on gender differences in vaccine uptake and acceptance has not provided consistent evidence of a significant difference. On the other hand, two groups of psychological factors were also found by the existing literature to be associated with COVID-19 vaccine acceptance and uptake. These factors include the perceptions of individuals about the seriousness and the threat of the virus (e.g., Dula et al., [9]; Gruner and Kruger, [14]) and the safety of COVID-19 vaccine (e.g., Machida et al., [7]; Soares et al., [10]; Chaudhary et al., [12]).

Despite the ongoing ramifications of the virus coupled with the very low vaccine uptake, there has been no available research concerning COVID-19 vaccine uptake and acceptance in Somaliland, thereby making this paper extremely essential at this point of time. The paper aims to bring light to the overall rate of vaccine uptake, willingness to get vaccinated, and the determinants of vaccine uptake and acceptance in Somaliland. The results of the study could help the government and other concerned parties shape policies that can boost vaccine uptake.

## 2. Data and Method

### 2.1. Sample and Data Collection

We carried out a cross-sectional survey with both offline and online modes using a structured questionnaire in the period from 18 December 2021, to 31 January 2022. To avoid language bias, the questionnaire was offered in both English and the local Somali language. Our survey covered adults in Somaliland who are 18 years and older. Participants of the online survey were recruited through a network of friends, researchers, and lecturers in Somaliland who shared the survey link across social media platforms. In addition, trained research assistants helped with the collection of the paper survey data. To assess the questionnaire validity, we have piloted the survey questionnaire and received feedback from multiple reviewers. The total responses we have received were 704 as presented in the Appendix A. However, due to missing data at the level of some variables, we have used 661 and 409 responses for the logistic regressions of the vaccine uptake and willingness, respectively.

### 2.2. Measurement of Variables

To operationalize the main dependent variable of the study, we asked respondents a dichotomous (yes/no) question of whether they have taken the COVID-19 vaccine. Independent variables, including socio-demographic factors, were measured following the prior literature. The key socio-demographic determinants of vaccine uptake included in our study were age (categorized into five groups in ascending order), gender, education (no education, primary, secondary, undergraduate, and postgraduate), region of residence (the six major regions of Somaliland), employment status (employed versus unemployed), and the respondent’s history of underlying health condition (i.e., heart disease, hypertension, diabetes, cancer, HIV/AIDS, or tuberculosis).

Following prior studies, we have considered indicators of two psychological factors that determine COVID-19 vaccine uptake or willingness to get vaccinated. First, we looked at the perceived threat of the virus (worry about the virus). Gruner and Kruger [14] show that concerns about the health threat of COVID-19 are significantly associated with the intention to get vaccinated using survey data from Germany. To measure the perceived threat, we asked respondents to rate whether COVID-19 poses a serious threat to their health on a 4-points Likert scale (strongly agree to strongly disagree). Second, a significant number of people in Somaliland were influenced by conspiracy theories on social media and other channels claiming that COVID-19 vaccines are not safe. We have noticed this from conversations we had with multiple individuals during the initial stage of designing this study. To measure the perceived safety of the vaccine, we asked respondents to rate the extent they agree with a statement on vaccine safety using a 4-point Likert scale. The detailed operationalization of study variables is presented in Table A1 in the Appendix A.

As the vaccines were not made available in all health centers in Somaliland, we recognize that the ease of access to COVID-19 vaccine may determine respondents’ vaccine uptake. In fact, Machida et al. [7] linked vaccine accessibility to higher vaccine uptake. We, therefore, included a question on whether the vaccines were made available at the workplace or at a nearby health center. We also control for respondent’s residence, questionnaire language, language mode, and social media consumption. To measure social media consumption, respondents were grouped into four categories based on the number of hours they spend on social media per day.

### 2.3. Statistical Analysis

We used descriptive statistics and group comparison tests to understand the sample of our study and provide a preliminary overview. Given that the dependent variable of the study is dichotomous, we employed a binary logistic regression to test the factors associated with vaccine uptake and willingness to get vaccinated.

## 3. Results

### 3.1. Descriptive Statistics

Participants of the survey were mainly males (60%), single (60%), in the age groups of 18–24 (42%) and 25–34 (34%), mostly healthy (84.9%), and highly educated, with 78% of them having a university degree. In addition, 60% of them are currently employed, with 15% of them reporting to work in the healthcare sector. The vast majority of the participants (91%) also resided in Maroodi Jeh region. Only 15% of the participants reported to suffer from at least one of the health conditions enlisted in the survey. Out of the 704 respondents we have surveyed, only 259 (37%) have taken the vaccine. Among those who were not vaccinated, 435 responded to our question about whether they have the intention to take the vaccine. About two-thirds of them were unwilling to take the vaccine (65%) as presented in Table 1.

### 3.2. Group Comparisons

Table 2 presents the comparative analysis of variables by groups. We used a Mann–Whitney test to examine the significance of difference between groups for dichotomous variables (i.e., gender, employment, healthcare, health condition, and availability). For all other variables, which are ordinal in nature, we utilized the Kruskal–Wallis test. Among socio-demographic factors, the age, education, and employment status of those vaccinated and those who were not vaccinated were significantly different (*p* < 0.01). Furthermore, the percentage of healthcare workers who were vaccinated were significantly higher than non-healthcare workers (*p* < 0.05). None of the socio-demographic factors were significantly different among the groups willing to get vaccinated. However, both of the vaccinated groups (or those willing to get vaccinated) and those who were not vaccinated (or not willing to get vaccinated) were different in how they perceived the threat of the virus and the safety of the vaccine (*p* < 0.01).

### 3.3. Regression Results

The results of the logistic regression in Table 3 (model 1) show that education and age are positively associated with vaccine uptake (*p* < 0.01). As seen earlier in Table 2, the vaccine uptake rate increased significantly as the age of the participants got older, with the uptake going from 27% in the 18–24 age group to 54% in those older than 55. Moreover, higher level of education was associated with higher vaccine uptake, with the rate ranging from 18% in those with no education to 49% in those with postgraduate education. Although those who were employed were more likely to get vaccinated (*p* < 0.01), as shown in Table 3 (model 1), our logistic regression test found no evidence of any difference in vaccine uptake and acceptance in those who are employed in the healthcare sector and other unrelated sectors (*p* > 0.1). With the very small number (15%) of survey participants who reported to work in the healthcare sector, our sample may not be representative of healthcare workers in Somaliland. It is also important to note that the results in Table 2 are bivariate comparison tests and do not control for other factors.

For psychological factors, both the perceived threat of the virus and perceived safety of the vaccine were strongly significant in the decision of the participants to take the vaccine (*p* < 0.01). Among control variables, we found that the ease of access to the vaccine was a key determinant of vaccine uptake (*p* < 0.01). In addition, respondents who chose the English version (versus the Somali version) were significantly more likely to get vaccinated (model 1 of Table 3) or to be willing to get vaccinated (model 2 of Table 3).

## 4. Discussion

To the best of our knowledge, this research on the uptake and acceptability of the SARS-CoV-2 vaccine is the first to be done in Somaliland. It estimates that a minor proportion of the population (37%) took the vaccine and only 35% of those that didn’t get vaccinated were willing to take it. Our results were contradictory to the acceptance rate reported by Ahmed et al. [6], who has found that more than three quarters (76.8%) of the Somali population were willing to take the vaccine once it is made available. Although there were similarities in the study population, it is important to firstly note that only 1.5% of the total participants in this study reported living in Somaliland. Secondly, it is possible that the intentions to take the vaccine among the Somali population changed over time. This is consistent with findings reported by Robinson et al. [7] who indicated that, as the pandemic progresses, intentions to take the vaccine decrease. Furthermore, lastly, with survey results showing the perceived safety of the vaccine to be an important determinant of both uptake and acceptance, it is plausible that the public might have been dissuaded from taking the vaccine by the reported COVID-19 vaccine side effects. Hence, the very low uptake and acceptance rate that is in contrast to the rate estimated. Similarly, the low level of uptake (39.4%) reported by Alemayehu et al. [15] in neighboring Ethiopia was found to be due to participants believing that vaccines cause blood clots.

Sociodemographic factors that make the participants hesitant, according to our findings, include young age, low educational level, and unemployment. Conspiracy theories run rampant in the country, with many spreading false claims about the COVID vaccine. Illiterates and those with low levels of education could be the victims of such misinformation which could cause them not to take coronavirus vaccination. As such, the dissemination of the correct information in Somali to these groups and engaging other stakeholders like the religious and community leaders in tackling the misinformation and encouraging vaccine acceptance is necessary. Hesitancy among the young age groups could be mostly rooted in the fact that they do not face dire health consequences from the virus. Though the vaccinated individuals could still spread the virus to others, the risk of transmission is less compared to the unvaccinated, as was reported by Shah et al. [16], in an observational study among healthcare workers and their households. With this unfortunate reality in mind, the importance of at least lessening transmission and keeping their community healthy must be emphasized for the youngsters to encourage them to take the vaccine.

Variables on the perceived threat of the virus and safety of COVID-19 vaccine has shown that those who have not taken the vaccine or who are unwilling to take the vaccine perceive the vaccines as not safe and the health threat of the coronavirus to be minimal. These results are consistent with previous studies that linked lower acceptance rates to groups who worry less about the virus and those who lack confidence in the safety of the vaccine (e.g., Machida et al., [7]; Dula et al., [9]; Soares et al., [10]; Chaudhary et al., [12]; Kruner and Kruger, [14]). The evidence is also consistent with the findings of Bahta et al. [17] and Jama et al. [18], who found that misinformation is generally linked to the lower uptake of other vaccines among the Somali diaspora in US and Sweden.

With the reality being contrary to this belief, survey respondents seem to be ill-informed about the serious health consequences of the virus and the otherwise safe COVID-19 vaccines. Raising their awareness by providing materials that can educate them on both the virus and the vaccine can increase the vaccine acceptance and uptake. It is also important to note that respondents who chose the English version of our questionnaire were more likely to take the vaccine, indicating that those familiar with the English language were more aware of vaccine importance than those who are not. This may be due to the fact that information regarding COVID-19 on different platforms including social media, international news outlets, and other sources of health information like the WHO were made available in English. Therefore, given the significance of the language, it is important to place more emphasis on the translation of COVID-19-related materials into Somali language.

## 5. Conclusions

Our research has only scratched the surface of this crisis and has shown that the coronavirus crisis is far from over, with this very low vaccine uptake (37%) and acceptance (35%) rate in Somaliland. Our findings point to the cruciality of carrying out mass awareness campaigns to dispel myths and educate people on the threat of the virus and safety of the vaccines to increase these rates.

However, with 78% of the participants being students with a tertiary level of education, 91% residing in Maroodi Jeh region, and the vast majority being healthy with no underlying health conditions, our results should be interpreted with caution. Research on this subject matter with a wider sample that is representative of the general Somaliland population is of utmost importance in order to further investigate the reasons for vaccine acceptance and hesitancy, as we are more than likely going to live with the coronavirus for the foreseeable future.

## Figures and Tables

**Table 1:** Vaccine uptake and willingness.

	Group	Obs.	% Vaccinated/Willing
**Vaccinated**	Yes	259	37%
	No	5	63%
**Willing to get vaccinated**	Yes	153	35%
	No	282	65%

**Table 2:** Group comparison tests.

Variable	Group	Vaccinated		Willingness	
		Observations (% Vaccinated)	Difference Test	Observations (% Willing)	Difference Test
**Gender**	Female	279 (34)	−1.186 (0.236)	181 (39)	1.493 (0.135)
	Male	425 (39)		254 (32)	
**Age**	18–24	296 (27)	20.979 *** (0.0001)	214 (33)	6.244 (0.182)
	25–34	240 (40)		140 (35)	
	35–44	77 (54)		33 (45)	
	45–55	49 (43)		28 (50)	
	>55	41 (54)		19 (21)	
**Education**	No education	55 (18)	23.599 *** (0.0001)	44 (34)	7.321 (0.120)
	Primary	28 (33)		19 (37)	
	Secondary	63 (36)		38 (37)	
	Undergraduate	355 (33)		232 (31)	
	Postgraduate	201 (49)		100 (46)	
**Employment**	Yes	416 (43)	−4.032 *** (0.0001)	231 (34)	0.516 (0.606)
	No	285 (28)		202 (36)	
**Healthcare**	Yes	68 (54)	−2.391 ** (0.017)	30 (37)	−0.412 (0.681)
	No	378 (39)		225 (33)	
**Health Condition**	Yes	106 (45)	−1.954 (0.051)	57 (33)	0.312 (0.755)
	No	598 (35)		378 (35)	
**Availability**	Yes	532 (86)	−4.456 *** (0.000)	306 (69)	0.582 (0.561)
	No	165 (71)		125 (72)	
**Perceived threat**	Strongly agree	272 (48)	22.438 *** (0.0001)	140 (42)	14.018 *** (0.0002)
	Agree	241(32)		161 (40)	
	Disagree	129 (28)		91 (23)	
	Strongly disagree	56 (25)		40 (15)	
**Perceived safety**	Strongly agree	165 (32)	5.485 ** (0.019)	110 (23)	7.798 *** (0.005)
	Agree	224 (33)		147 (36)	
	Disagree	207 (44)		114 (46)	
	Strongly disagree	100 (39)		60 (35)	

Notes: Diff. Tests are group comparison tests. These are Mann–Whitney tests for binary variables and Kruskal–Wallis tests for other variables; figures between parentheses in difference test columns are p-values. Significance levels are at 1% (***), 5% (**) and 10% (*).

**Table 3:** Logistic regression analysis.

	Model 1: Vaccine Uptake			Model 2: Vaccination Willingness		
	Coeff.	Std. Error	Z-Test	Coeff.	Std. Error	Z-Test
**Gender**	−0.236	0.197	−1.20	−0.269	0.234	−1.15
**Age**	0.497 ***	0.090	5.30	0.254 **	0.129	1.99
**Education**	0.364 ***	0.108	3.36	0.237 *	0.135	1.75
**Employment**	0.423 **	0.216	1.96	−0.315	0.255	−1.24
**Health condition**	0.279	0.279	1.00	−0.391	0.378	−1.04
**Perceived threat**	0.522 ***	0.104	5.02	0.520 ***	0.134	3.88
**Perceived safety**	0.277 ***	0.093	2.96	0.335 ***	0.115	2.91
**Availability**	0.820 ***	0.230	3.56	−0.184	0.246	−0.75
**Residence**	−0.077	0.110	−0.70	0.103	0.118	0.78
**Language**	−0.577 ***	0.197	−2.92	−0.509 **	0.260	−1.96
**Mode**	0.207	0.199	1.04	−0.089	0.247	−0.36
**Social media use**	−0.051	0.100	−0.50	−0.024	0.118	−0.20
Cons	−3.731 ***	0.677	−5.51	−1.483 *	0.804	−1.85

Note: Model 1 (observations = 661; LR Chi2 = 125.61; *p*-value = 0.000; Psuedo R2= 14.43%). Model 2 (observations = 409; LR Chi2 = 37.39; *p*-value = 0.000; Psuedo R2 = 7.08%). Significance levels are at 1% (***), 5% (**) and 10% (*).

**Figure A1:**
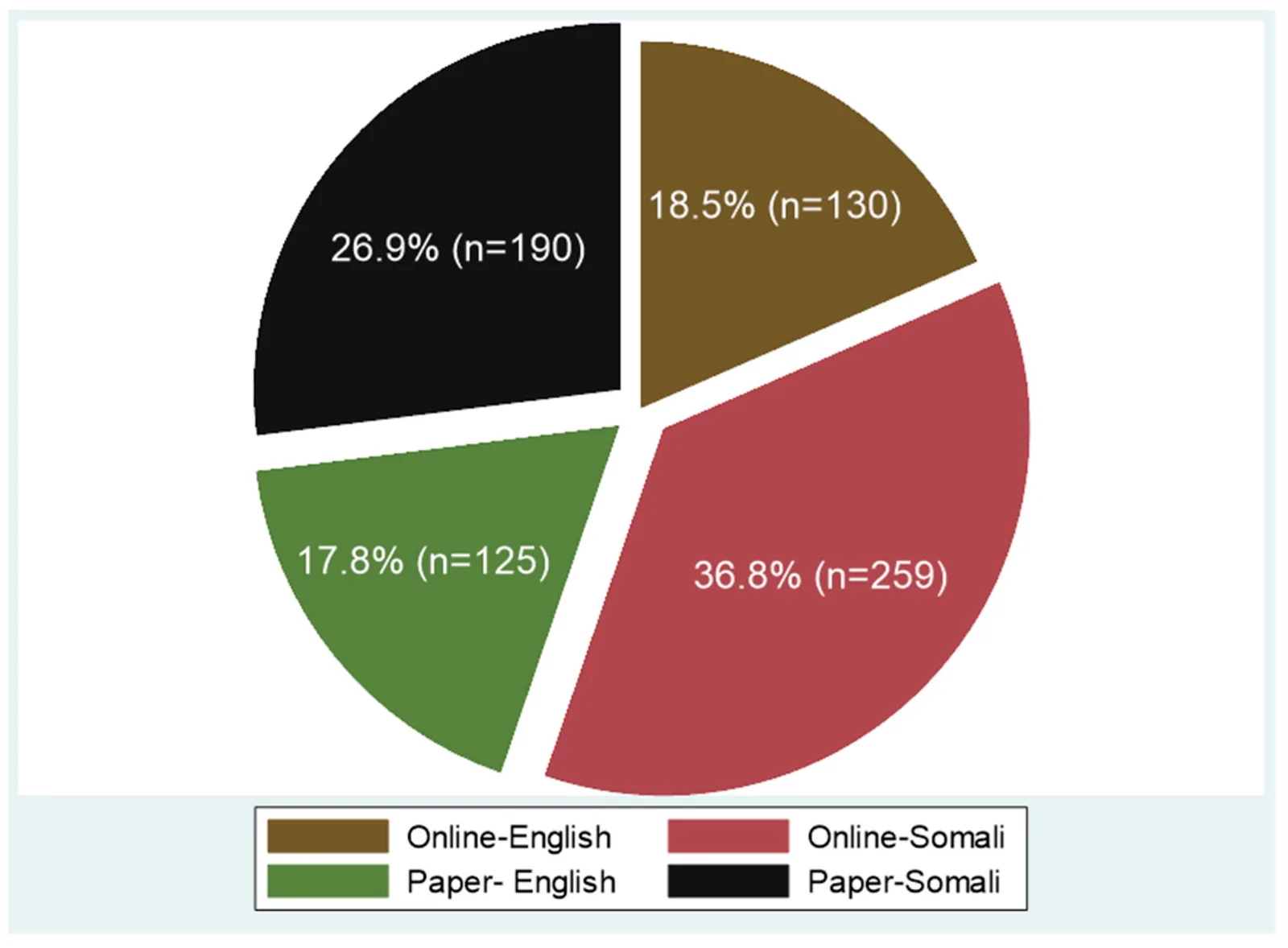
Data Collection Mode and Language.

## Data Availability

Data used in this study were obtained from a survey. The data are available at https://data.mendeley.com/datasets/56hg54ww63/1 (accessed on 20 June 2022).

## Appendix A

**Table A1:** Measurement of variables.

Variable	Question/Statement	Code
Vaccine uptake	Have you taken the COVID-19 vaccine?	1 = yes, 0 = no
Willingness	Are you willing to take the vaccine?	1 = yes, 0 = no
Gender	Participants gender	0 = female, 1 = male
Age	Age group	0 = 18–24, 1 = 25–34, 2 = 35–44, 3 = 45–55, 4 = older than 55
Education	Level of education	0 = no education, 1 = primary, 2 = secondary, 3 = undergraduate, 4 = postgraduate
Marital status	Marital status	0 = single, 1 = married, 2 = divorced, 3 = widowed
Employment	Are you currently employed?	1 = yes, 0 = no
Health condition	Have you ever been diagnosed with any of the following underlying health conditions (Choose all that apply)?: heart disease, hypertension, diabetes, cancer, HIV/AIDS, Tuberculosis).	1 if the respondent chooses at least one of the underlying health conditions specified, 0 otherwise
Availability	Was the vaccine made available at work/study place or at a nearby health center?	1 = yes, 0 = no
Perceived threat	COVID-19 poses a serious threat to my health	3 = strongly agree, 2 = agree, 1 = disagree, 0 = strongly disagree
Perceived vaccine safety	COVID-19 vaccine is not safe	3 = strongly disagree, 2 = disagree, 1 = agree, 0 = strongly agree
Residence	Region of residence in Somaliland	1 = Maroodi Jeeh, 2 = Togdheer, 3 = Sahil, 4 = Awdal, 5 = Sool, 6 = Sanaag
Language	Language of questionnaire chosen	1 = English, 2 = Somali
Mode	Online survey vs. paper survey	1 = paper survey, 2 = online survey
Social media use	Approximately, how many hours do you use social media per day?	0 = less than 2 h, 1 = 2 to 4 h, 2 = 5 to 7 h, 3 = more than 7 h
